# “HEATPAC” - a phase II randomized study of concurrent thermochemoradiotherapy versus chemoradiotherapy alone in locally advanced pancreatic cancer

**DOI:** 10.1186/s13014-017-0923-8

**Published:** 2017-11-21

**Authors:** Niloy Ranjan Datta, Bernhard Pestalozzi, Pierre-Alain Clavien, Alexander Siebenhüner, Emsad Puric, Shaka Khan, Christoph Mamot, Oliver Riesterer, Jürg Knuchel, Cäcilia Sophie Reiner, Stephan Bodis, members of the HEATPAC Trial Group, Michelle De Oliveira, Henrik Petrowsky, Thomas Winder, Matthias Guckenberger, Stephanie Lang, Peter Bauerfeind, Beata Bode, Oliver Tschalèr, Dietmar Marder, Thomas Roeren, Rainer Grobholz, Burkhardt Seifert

**Affiliations:** 1 Centre for Radiation Oncology KSA-KSB, Kantonsspital Aarau AG, Tellstrasse, CH-5001 Aarau, Switzerland; 2 Centre for Hematology and Oncology, University Hospital Zurich, Zürich, Switzerland; 3 Department of Surgery and Transplantation, University Hospital Zurich, Zürich, Switzerland; 4 Centre for Medical Oncology / Hematology, Kantonsspital, Aarau, Switzerland; 5 Department of Radiation Oncology, University Hospital Zurich, Zürich, Switzerland; 6 Department of Gastroenterology and Hepatology, Kantonsspital, Aarau, Switzerland; 7 Institute for Diagnostic and Interventional Radiology, University Hospital Zurich, Zürich, Switzerland; 8 Members, Associated departments of Kantonsspital Aarau and University Hospital Zurich, Zürich, Switzerland

**Keywords:** Pancreatic cancers, Hyperthermia, Radiotherapy, Chemotherapy, Randomized trial, Study protocol

## Abstract

**Background:**

Pancreatic cancer has a dismal prognosis with 5-year overall survival rate of around 5%. Although surgery is still the best option in operable cases, majority of the patients who present in locally advanced stages are deemed inoperable. Novel approaches are therefore needed for the management of around 80% of these inoperable locally advanced pancreatic cancers (LAPC). Hyperthermia (39–43 °C) is a potent radiosensitizer and further enhances the action of gemcitabine, also a known radiosensitizer. Thus through triple sensitization, a combination of hyperthermia, radiotherapy and gemcitabine could be expected to improve the therapeutic outcomes in LAPC.

**Methods:**

This phase II randomized trial, HEATPAC in unresectable LAPC, explores the feasibility and efficacy of concurrent thermochemoradiotherapy (HTCTRT) over chemoradiotherapy (CTRT) alone with pre- and post-intervention FOLFIRINOX at standard dosage and schedule. Following 4 cycles of neoadjuvant FOLFIRINOX, patients with no metastasis and absence of gross peritoneal carcinomatosis would be randomized to either (a) control arm: concurrent CTRT with gemcitabine (400 mg/m^2^, weekly ×6) or (b) study arm: locoregional hyperthermia (weekly ×6 during radiotherapy) with concurrent CTRT (same as in control arm). All patients would receive simultaneous-integrated boost intensity-modulated radiation therapy to doses of 56Gy and 50.4Gy to the gross and clinical target volumes respectively delivered in 28 fractions over 5.5 weeks. Deep locoregional hyperthermia would be administered weekly and monitored with real-time intraduodenal multisensor thermometry probe. A temperature of 40–43 °C for 60 min would be aimed for each hyperthermia session. On completion of CTRT/HTCTRT, patients of both groups would receive an additional 8 cycles of FOLFIRINOX.

**Discussion:**

The expected 1-year baseline overall survival with CTRT alone is considered as 40%. With HTCTRT, a survival advantage of +20% is expected. Considering α = 0.05 and β = 0.80 for sample size computation, a total of 86 patients would be equally randomized into the two treatment groups. This phase II study if found to be safe and effective, would form the basis of a future phase III randomized study.

**Trial registration:**

The trial has been registered with the *ClinicalTrials.gov* (NCT02439593). The study has been approved by the Ethical Commissions of Basel and Zurich and is open for patient recruitment.

## Background

### Locally advanced pancreatic cancers: a lethal disease

Worldwide pancreatic cancer is the 7th leading cause of death and has a fatal prognosis with 5-year survival rate of less than 5% [1–5]. Globally around 340,000 cases are diagnosed each year. As per the GLOBOCAN 2012, the incidence and the mortality rates of pancreatic cancers even in Europe are nearly similar at 6.8 and 6.6 per 100,000 respectively, indicating that nearly all diagnosed pancreatic cancers carry a lethal prognosis [5, 6]. The fatal consequences of pancreatic cancers is aptly summarized by D. Schrag in a recent editorial in JAMA as, “*If cancer is the emperor of all maladies, then pancreatic adenocarcinoma is the ruthless dictator of all cancers*” [2].

Despite advances in chemo-radiation and advent of newer systemic agents, outcome in patients with primary pancreatic adenocarcinoma has not changed significantly over the last decades. Complete surgical resection is still the only curative therapeutic option. However, at diagnosis, merely 10–20% of patients are candidates for surgical resection, with only 4% expected to undergo radical surgery. 40% of the patients are known to present in advanced stages. Despite the best combination chemotherapy (CT) or chemoradiotherapy (CTRT), 30% of these patients die due to local progression of disease without any distant metastasis [1–4].

### Possible therapeutic approaches in locally advanced pancreatic cancers

CT alone or CTRT has been generally accepted as the standard therapeutic approach for patients with locally advanced pancreatic cancers (LAPC). In those without metastasis, neoadjuvant CT before a definitive CTRT has been used [1–4]. The role of CTRT was initially defined by Gastrointestinal Tumor Study Group (GITSG) [7]. In this study, the combination of bolus 5-fluorouracil (5-FU) and split course radiation (RT) (total dose, 4000 cGy) was compared with RT alone or with 6000 cGy combined with 5-FU. A nearly 2-fold increase in median survival (42.2 versus 22.9 weeks) was observed with the regimen of bolus 5-FU and 4000 cGy compared to RT alone.

Subsequent generations of studies have sought to optimize the use of 5-FU and most contemporary studies no longer use split-course radiation. Gemcitabine has also been used as a radiosensitizer and there is evidence to suggest that concurrent gemcitabine and RT can yield similar or better outcomes when compared to 5-FU based CTRT [8]. A number of combination CT regimens with or without gemcitabine have been tried in LAPC. A recent systematic review with network meta-analysis of 9989 patients from 23 studies involving 19 different regimes have demonstrated a statistically significant improvement in overall survival (OS) and progression free survival (PFS) relative to gemcitabine [9]. The four drug regimen of folinic acid, oxaliplatin, irinotecan and 5-FU (FOLFIRINOX) appears to be the most favored combination with significant improvement in OS and PFS compared to most of the other combinations.

Chen et al. [10] undertook a meta-analysis to evaluate the long-term clinical efficacy of CTRT over RT or CT alone in LAPC. Their analysis consisted of data synthesized from 15 eligible randomized controlled trials of a total of 1128 patients. Of the 15 trials, 12 reported 6-month survival (*n* = 964), 14 (*n* = 1098) reported 12-month survival while 9 (*n* = 805) reported survival at 18-months. The meta-analysis showed that the CTRT group had a superior survival over the CT or RT group while the 18-month survival showed no significant difference. Patients on CTRT had a significantly higher grade 3–4 treatment related hematological and non-hematological toxicities than those on CT or RT alone. Thus, the meta-analysis indicates that CTRT is more efficacious than RT or CT alone and provides an improvement in OS for patients of LAPC, but at the cost of increased grade 3–4 toxicities.

In a bid to improve the outcomes in LAPC, erlotinib, a reversible tyrosine kinase inhibitor acting on the epidermal growth factor (EGFR) has been also tried along with CT (gemcitabine). In a recently reported outcome of the LAP07 international, open label, phase III randomized study, participants were first randomized to gemcitabine (*n* = 223) or gemcitabine with erlotinib (*n* = 219). After 4 months of treatment, patients who were free of any disease progression, underwent a second randomization to either continue their CT (*n* = 136) or begin CTRT (*n* = 133) [4]. There was no significant difference in the overall survival with CTRT compared with CT alone, nor with gemcitabine versus gemcitabine plus erlotinib used as maintenance therapy. Moreover, patients receiving erlotinib experienced added toxicities. A recently reported systematic review and meta-analysis of 27 randomized studies consisting of 8205 patients for targeted therapy for anti-EGFR showed that on pooled analysis, no significant benefit could be demonstrated in response rates, OS or PFS with targeted-based therapies as compared to conventional treatments [11]. Thus, even the molecular targeted therapies could not get translated into a better therapeutic benefit in LAPC.

### Hyperthermia in combination with chemoradiotherapy as a therapeutic option

Hyperthermia (HT) at 39–43 °C is a potent radiosensitizer and synergistic to a number of chemotherapeutic agents, like gemcitabine [12, 13]. In additional to the various thermoradiobiological interactions that accords a sensitizing effect to HT, heat also activates dendritic cells in conjunction with RT or CT. The tumor antigens derived from the necrotic tumor cells could be taken up by dendritic cells leading to heat shock protein (HSP) mediated induction of immunogenic tumor cells resulting in immunomodulation, a phenomena mimicking HT induced "*in situ tumor vaccination"* [14]. In addition, gemcitabine is also a known radiosensitizer due to (a) reduced RT induced DNA repair (b) “S” phase block and (c) triggering of apoptosis [15, 16]. Furthermore, HT has been shown to sensitize the effects of gemcitabine at 43 °C. This has been best observed if gemcitabine is given 24 h after HT [17].

Based on the thermoradiobiological basis of the interaction of HT, RT and CT, a number of phase I/II pilot studies have been undertaken by various institutions. Most of them have shown improved outcomes with the above modalities incorporating HT without any significant added morbidity or mortality. Patient tolerance has been reported to be satisfactory [18–22]. In one of the largest series of 68 patients, 34 evaluable patients were treated with HT added to CTRT (HTCTRT), while 26 received CTRT alone [18]. At a follow-up of 12-months, 22/34 patients (64.7%) in the HT group and 16/26 patients (61.5%) in the CTRT group were still alive. The median OS was 15 months (range: 6–20 months) in the HT group versus 11 months (range: 5–13 months) in the control group (*p* = 0.025). Median time to progression was 11 months (range: 3–16 months). As the study was a nonrandomized trial and based on patient preferences, the significant overall survival advantage reported with HTCTRT over CTRT alone needs cautious interpretation.

A review of the EU Clinical Trials site reveals a list of 109 trials that are currently being investigated by various centers for “locally advanced pancreatic cancers” [23]. A wide range of treatment options using gemcitabine, FOLOFIRINOX, other chemotherapeutic agents as primary therapy, second line / salvage, neoadjuvant, concurrent with radiotherapy and adjuvant therapy are under various phase I to III trials. Only one study is listed in which HT is being explored with gemcitabine and cisplatinum in patients with locally advanced or metastatic pancreatic cancer as second-line therapy after adjuvant or first-line chemotherapy with gemcitabine or gemcitabine combination (EudraCT Number: 2005–003855-11). The study was activated in June 2006, but the outcomes are not yet available.

Presently a phase III trial (EudraCT Number: 2008–004802-14), of adjuvant gemcitabine and capecitabine versus gemcitabine with cisplatin along with local HT is ongoing in patients of resectable pancreatic cancers following a R0 or R1 resection. This trial, “Hyperthermia European Adjuvant Trial (HEAT)” aims to look for differences in outcomes following HT added to either of these two CT regimens [24]. The study is currently open and recruiting patients. It is a multi-centric trial being conducted in various centers of Europe, primarily in Germany. A total of 366 patients is planned to be randomized into these two groups following R0/R1 resection. The endpoint to be evaluated includes OS and PFS.

Thus, as the HEAT study is being conducted in resectable pancreatic cancers to explore the likely advantage of HT added to adjuvant CT, the present HEATPAC study is primarily for unresectable LAPC. Since most of the patients of pancreatic cancers in locally advanced stages are deemed inoperable, it would be of considerable interest to explore the safety and efficacy of HTCTRT (with concurrent gemcitabine) compared to CTRT alone with pre- (as neoadjuvant) and post-intervention FOLFIRINOX. The use of HT, RT and gemcitabine is expected to provide triple sensitization with HT and gemcitabine both as radiosensitizer and additionally HT as a sensitizer to gemcitabine. This forms the rationale of the proposed phase II randomized study (HEATPAC), comparing HTCTRT versus CTRT in LAPC [25].

## Methods and design: HEATPAC study protocol

### Study design

**Fig. 1:**
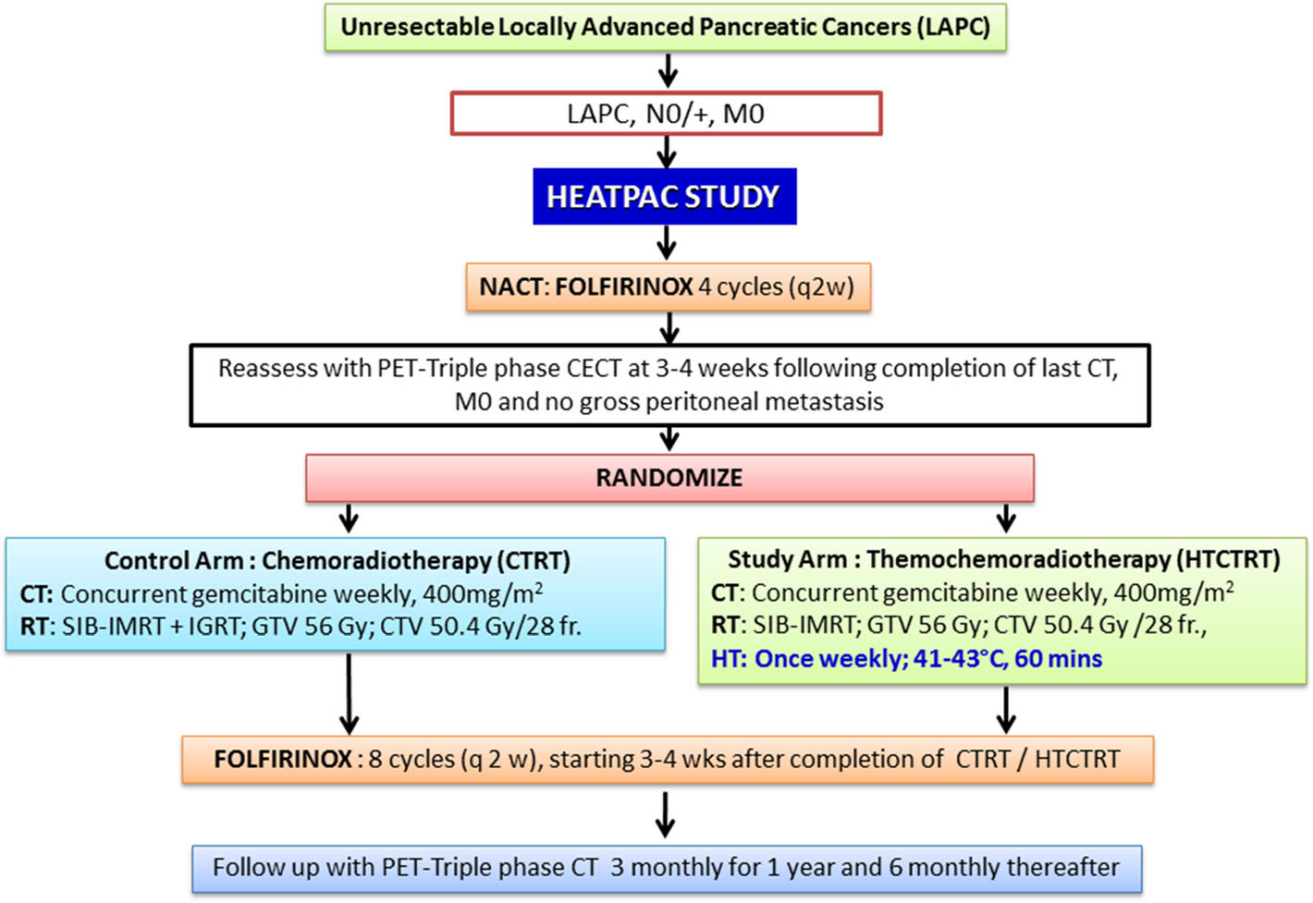
Flow chart of the HEATPAC study protocol. (CT: chemotherapy; RT: radiotherapy; HT: hyperthermia; NACT: neoadjuvant chemotherapy; SIB-IMRT: simultaneous-integrated boost intensity-modulated radiotherapy; IGRT: image guided radiation therapy; GTV: gross target volume; CTV: clinical target volume; FOLFIRINOX: calcium folinate, oxaloplatin, irinotecan, 5-FU)

This is a phase II randomized study in LAPC (Fig. 1). Patients of pancreatic cancers fulfilling the following criteria of “unresectable LAPC” would be considered for this trial. These include, presence of solid tumor contact with superior mesenteric artery or coeliac axis of >180°, aortic involvement, unreconstructible superior mesenteric vein or portal vein due to tumor involvement or occlusion due to tumor or thrombus [26, 27]. All patients who following four cycles of neoadjuvant FOLFIRINOX on a PET-triple phase CECT are free of any distant metastasis or gross peritoneal carcinomatosis would be randomized to either:

- Control group: CTRT with concurrent gemcitabine and RT or.
- Study group: HTCTRT with concurrent gemcitabine, RT and local HT.

### Primary endpoints

1. To determine if HTCTRT following neoadjuvant and adjuvant CT with FOLFIRINOX would show an improvement of OS at 1 year from 40% to 60% compared to concurrent CTRT alone.
2. To assess the acute and the late morbidities associated with HTCTRT compared to concurrent CTRT alone.

### Secondary endpoints

To compare the progression free survival, local disease free survival, disease free survival in patients treated with HTCTRT versus CTRT.

### Inclusion criteria

1. Patients of unresectable LAPC as defined above [26, 27].
2. Histopathologically proven ductal adenocarcinoma of the pancreas (biopsy/cytology).
3. ECOG performance scale 0 and 1.
4. Age between 18 and 80 years.
5. Absence of distant metastasis or gross peritoneal carcinomatosis.
6. Adequate kidney functionality defined as creatinine clearance >50 ml/min.
7. Adequate liver functionality defined as total bilirubin ≤2 times of the upper limit of normal.
8. Adequate bone marrow reserves: WBC count ≥2.5 × 10^9^/L, platelet count ≥100 × 10^9^/L, hemoglobin ≥8.0 g/L.
9. Women of child-bearing age must secure sufficient contraception control (dual protection with condoms and pills) during the clinical trial and 6 months after the clinical trial is completed.
10. For females of child bearing potential, pregnancy test within 2 week prior to randomization should be negative.
11. Absence of psychological, familial, sociological or geographical condition that could potentially hamper compliance with the study protocol and follow-up schedule.
12. Ability to travel to Kantonsspital Aarau for weekly HT. RT and CT could be administered both at University Hospital Zurich or Kantonsspital Aarau or other participating institution as per the patient’s preference.

### Exclusion criteria

1. Prior or concurrent malignancies.
2. Patients having metal implants, pacemakers or clustered markers.
3. Patients with metallic endobiliary stent would need to be replaced with plastic stents.
4. Any history of myocardial infarction within the past 12 months.
5. Any connective tissue disorder that contraindicate RT, e.g., scleroderma.
6. Pre-existing grade 2 peripheral neuropathy.
7. Any known contraindication or hypersensitivity to the chemotherapeutic agents used in the study as decided by the medical oncologists.
8. Pregnancy, lactation period or lack of reliable contraception.
9. Any other disease or therapy, which, according to the investigator, present a risk to the patient or which are not compatible with the aims of the clinical trial.
10. Indications that the person concerned will possibly not keep to the clinical trial plan because of unwillingness to cooperate or difficulties in keeping the check-up appointments.

### Study interventions: Chemotherapy

All LAPC patients would be receiving neoadjuvant FOLFIRINOX. It would consist of a 2-h intravenous infusion of oxaliplatin 85 mg/m^2^, followed by a 2 h intravenous infusion of calcium folinate 350 mg/m^2^ concomitantly with 90 min of intravenous infusion of irinotecan 180 mg/m^2^, subsequently followed by 5-FU 400 mg/m^2^ as a bolus and 2400 mg/m^2^ as a 46 h continuous intravenous infusion. All patients would routinely receive ondansetron and dexamethasone with each cycle for emesis prophylaxis. A total of 4 cycles at 2 weekly intervals would be administered as neoadjuvant CT. Eight such cycles would be also administered after the completion of CTRT or HTCTRT. Following the completion of 4 cycles of neoadjuvant FOLFIRINOX, patients would be randomized to either CTRT or HTCTRT provided there is no evidence of distant metastasis or gross peritoneal carcinomatosis on PET-triple phase CECT scan.

**Fig. 2:**
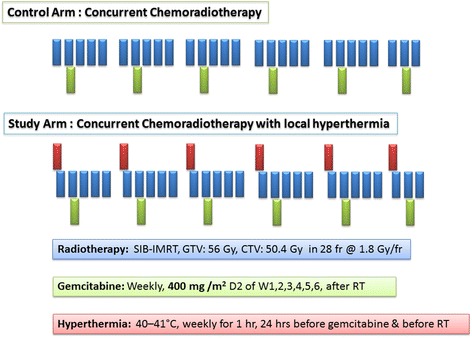
Schematic representation of the study and control arms with chemotherapy, radiotherapy and hyperthermia. (RT: radiotherapy; SIB-IMRT: simultaneous-integrated boost intensity-modulated radiotherapy; GTV: gross target volume; CTV: clinical target volume)

During RT, all patients would receive concurrent gemcitabine at 400 mg/m^2^, delivered intravenously over 30 min on day 2 of week 1 to 6 of RT (Fig. 2). Patients would be closely followed up for any adverse effects and dose modification would be made at the discretion of the treating physician based on observed toxicity.

### Study interventions: Radiotherapy

RT protocols to be followed in this study have been adapted as per the Radiation Therapy Oncology Group (RTOG) protocols, RTOG 0848 and RTOG 1201, wherever applicable [28, 29]. All patients in both arms would be planned with simultaneous-integrated boost intensity-modulated radiation therapy (SIB-IMRT) with 6MV photon or higher and assisted with image guided radiotherapy. The prescribed RT dose to the 95% of the gross target volume (GTV) would be 56Gy and 50.4Gy to the 95% of the clinical target volume (CTV). IMRT would be delivered 5 days a week at 1.8Gy per fraction. The maximum dose (Dmax) allowed within the GTV and CTV to a point that is 0.03cm^3^ would be 110% of the prescribed dose to these target volumes provided that the normal-tissue constraints are met. The minimum dose (Dmin) to the planning target volume (PTV) to a point, 0.03cm^3^ would be 95% of the prescribed dose to these target volumes.

The GTV would consist of primary tumor plus any regional lymph nodes identifiable on CT / MRI or on PET-CECT scan. The CTV would be based on the review of Sun et al. [30] where each lymph nodal region with risk of involvement of >3% was considered to be a clinically significant risk and proposed to be considered as an elective nodal irradiation area. Planning target volume (PTV) would be defined separately for GTV (PTV1) and CTV (PTV2). PTV1 will be GTV plus 0.5 cm in all directions while PTV2 would be CTV plus 0.5 cm in all directions when breath-hold, gating or tracking techniques are used. With free breathing, CTV to PTV expansion in the cranio-caudal direction will be based on target motion as assessed by 4D CT scan (but not exceed 1.5 cm). Expansions in other directions will be 0.5 cm. The normal tissue dose constraints as adapted by RTOG 1201 would be considered for this study [29].

### Study interventions: Hyperthermia

HT would be delivered at Kantonsspital Aarau, Aarau, Switzerland and regular quality assurance would be carried out in accordance with European Society of Hyperthermia Oncology (ESHO) quality assurance guidelines for clinical studies in regional deep HT [31, 32]. Deep HT would be administered by the BSD 2000 unit with the Sigma-60 or Sigma-Eye phased array applicator (M/s Pyrexar Medical, Salt Lake City, Utah, USA).

**Fig. 3:**
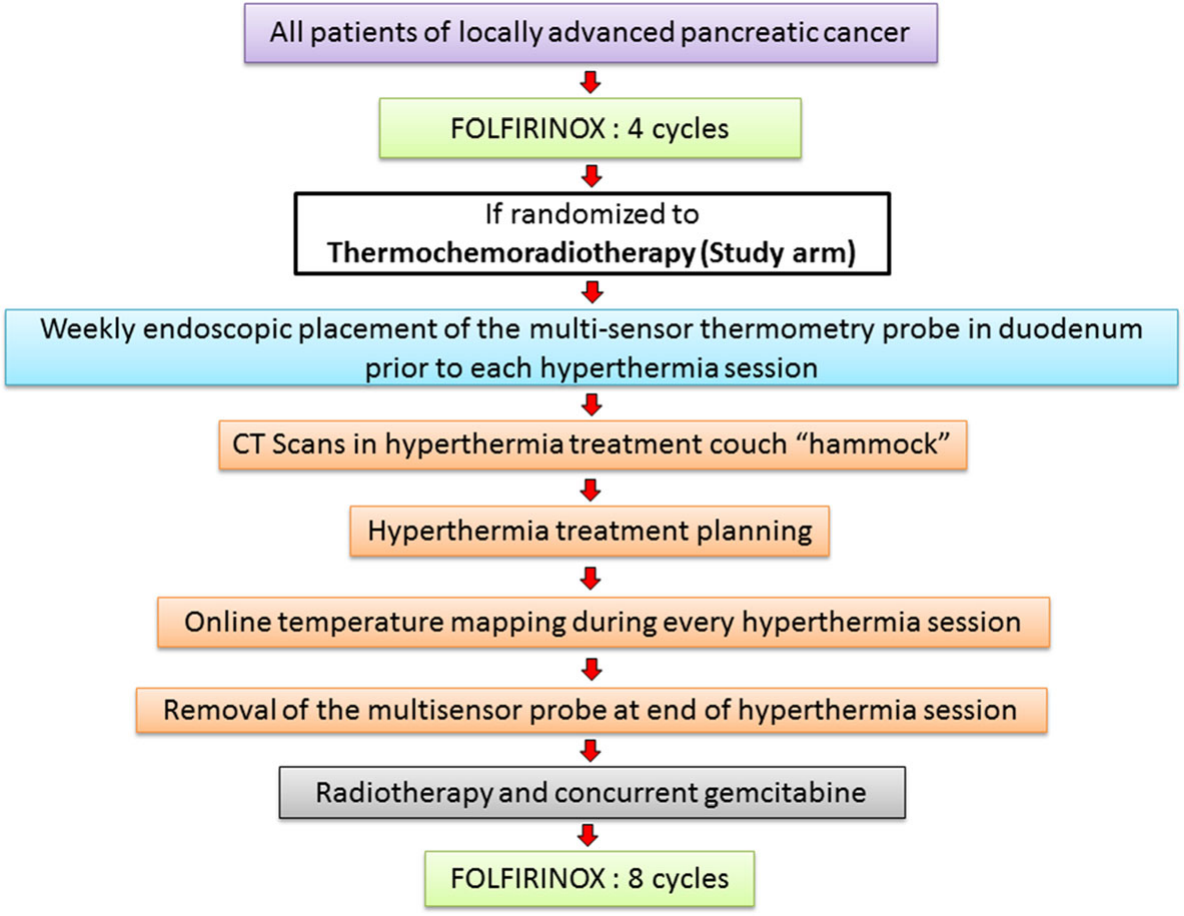
Workflow of the HEATPAC study protocol for patients’ to be treated with thermochemoradiotherapy. (FOLFIRINOX: calcium folinate, oxaloplatin, irinotecan, 5-FU)

To define the HT treatment volume with deep hyperthermia unit, CT would be carried out in HT treatment position on the HT planning support (hammock) adapted for CT. Radiographic markers would be positioned as for the RT planning CT to define a reference point, which would be visible in the planning system. The tumor and the adjacent normal structures would be contoured in these scans and attempt would be made to have a similar outline of the target and organs at risk as in the photon RT plan. The HT treatment planning target volume would be chosen typically based on the radiotherapy PTV/CTV and planned using the HT treatment planning, Sigma HYPERPLAN software (M/s Dr. Sennewald Medizintechnik GmbH, Munich, Germany) by segmentation and creation of a grid model of the various body tissues according to their dielectric properties (e.g., tumor, intestine, abdominal organs, muscle, bone, fat) followed by simulation of the electric fields. The Sigma HYPERPLAN, version 2.0, has specific perfusion factors for each tissue type that takes into account the blood circulation in the tissue (Fig. 3). The above structures would be defined individually during the contouring and thermal treatment planning. Using appropriate power and steering parameters, a specific absorption rate distribution in the target volume would be generated using finite element modeling. A warm-up heating phase of 30 min followed by 60 min of HT treatment would be used.

As per the ESHO guidelines for thermal mapping and to ensure quality assurance in HT treatment [31, 32], prior to each HT treatment, a multi-sensor thermometry probe (FISO, FISO Technologies Inc. Quebec, Canada) would be inserted endoscopically to cross beyond the duodenum such that the temperature measuring sensors are along the duodenal part encircling the pancreas. The probe having a length of 115 cm has a diameter of 870 μm and fits into a 6F catheter. The 8 sensors are spaced at every 2 cm which would be used for online temperature monitoring. Check films would be taken to verify the position of the temperature probe. Attempts would be made to attain a temperature of 40–43 °C taking into consideration individual patient tolerance to hyperthermia (Fig. 3). Temperature mapping would be carried out with a mapping interval of 5–10 min. The total treatment period would consist of the heating period (until the target temperature is reached, maximum 30 min) and thereafter the therapy period (target temperature maintained for 60 min).

Patients would be instructed to mention any unpleasant sensation suggesting a hot spot, such as a burning sensation, a feeling of pressure, or any pain. By switching off the power briefly (30 s), it would be established if the pain was caused by the radiated power. Accordingly the treatment settings (phase and amplitude) would be adjusted, or water cooling bags applied for complaints on the skin surface. For pain caused by the tumor or positioning in the applicator, analgesics might be given. Following each HT treatment, the thermometry probes would be removed. The procedure would be repeated for each weekly HT sessions during the entire course of treatment.

### Evaluation of endpoints

All patients would be closely monitored during the entire course of the study for any potential adverse event. All adverse events either observed by the physician and/or reported by the patient will be documented as per the “Common Terminology Criteria for Adverse Events” (CTCAE) guidelines, version 4.03 [33].

Response to SIB-IMRT and HT will be carried out by comparing tumor size at the time of registration for the study with measurements taken 4 weeks after completion of RT. Response will be measured through radiological imaging by triple phase CECT. MRI with or without PET-CT would be optional, but not used for loco-regional response scoring. The response would be evaluated as per the revised “Response Evaluation Criteria in Solid Tumors” (RECIST, version 1.1) [34].

The survival end points would be evaluated during the entire study period. Overall survival represents the time from date of registration (at the point of randomization) to the time of death from any cause during the entire study period. Patients surviving at the end of study period would be considered as “survivors” for computation of the overall survival. Death from any cause would be considered as an “event” for Kaplan-Meier estimations. Patients will be followed until the study ends (4.5 years from date of approval from Ethical Commissions). The other evaluable secondary endpoints would include progression free survival, local disease free survival and disease free survival. At the conclusion of the study, the survival endpoints would be evaluated by univariate and multivariate analysis to look for any potential risks and predictive parameters.

### Sample size considerations

**Fig. 4:**
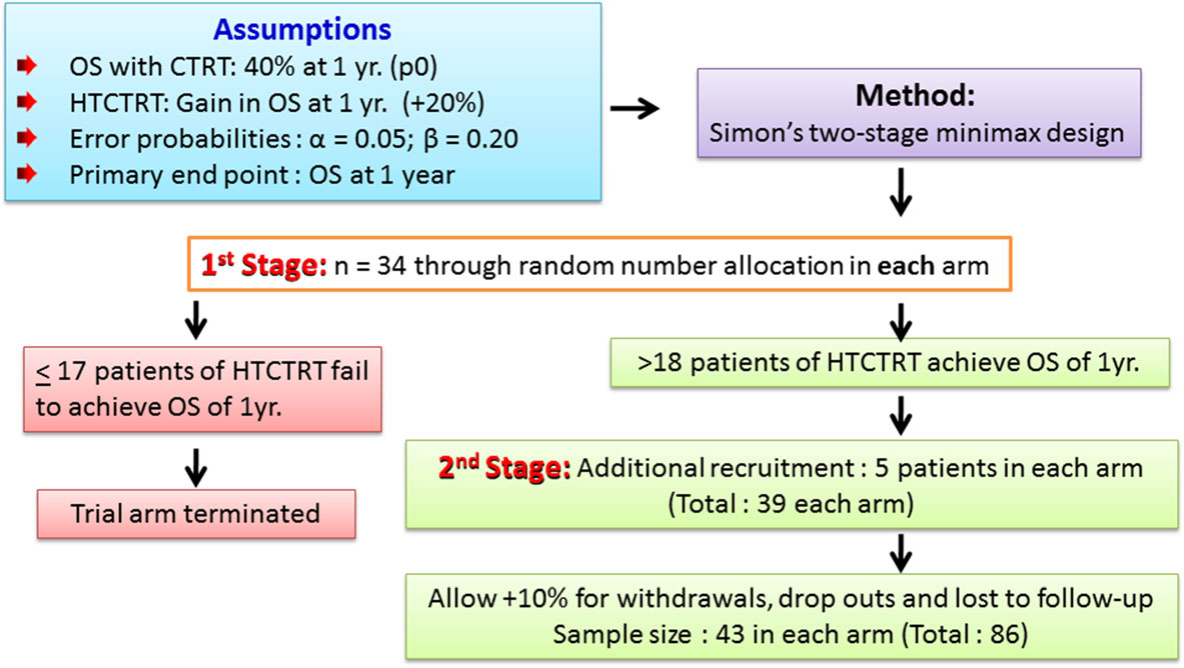
Sample size calculations for the HEATPAC study as per the Simon’s two-stage minimax design [35]. (OS: overall survival; CTRT: chemoradiotherapy; HTCTRT: thermochemoradiotherapy)

One-year overall survival (death from any cause) would be considered as the primary endpoint for the study. The period of survival would be computed from the date of randomization into the HEATPAC protocol to the last follow up or date of death from any cause. The 1-year survival rate with CTRT is considered as 40% (p0 = 40%). With HTCTRT, an overall survival advantage of +20% is expected (p1 = 60%). The sample size calculations were based on Simon’s two-stage minimax design [35] with α = 0.05 and β = 80%. Assuming a drop-out rate of 10%, a total of maximum 86 patients would be divided in the 2 groups of HTCTRT and CTRT of 43 patients each (39 + 4) (Fig. 4).

### Trial registration, ethical and legal considerations

The study is registered with the *ClinicalTrials.gov* (NCT02439593) and has been approved by the Ethical Commissions of Basel and Zurich, Switzerland [25]. All patients enrolled for the study would be required to furnish an informed consent. The responsible investigator will ensure that this study is conducted in agreement with the Declaration of Helsinki and conducted according to the ICH Harmonized Tripartite Guideline for Good Clinical Practice. Patient confidentiality would be maintained and the patient data would be available only to nominated co-investigators of the HEATPAC study group. During the course of the study the investigator’s site will be visited periodically and relevant documents would be available for review by the independent monitors of the trial.

### Sponsorship

The study has been jointly sponsored by the Radiation Oncology Centre KSA-KSB, Kantonsspital Aarau, Switzerland and Clinic for Oncology, University Hospital Zurich, Switzerland.

## Discussion

Following the demonstrated biological rationale of hyperthermia, there has been resurgence of using HT in clinics. HT unlike other forms of anticancer therapies (like RT and CT), is well tolerated, safe and without any significant acute or late toxicities. It’s one of the most potent radiosensitizer and also exhibits thermal synergism to a number of CT agents. HT is thus a unique therapeutic modality and has been shown to improve the therapeutic outcomes in a wide range of malignancies. These include - locally recurrent breast cancers, locally advanced head and neck cancers, locally advanced cancer cervix, soft tissue sarcoma, melanoma, pelvic tumors and others [12, 36–39]. Further, HT has also been shown to be a potential immunomodulating agent when used with RT [14]. It therefore certainly deserves to be explored along with the standard treatment modalities of CT and RT, especially in those disease sites where the standard therapeutic approaches of CT and RT have failed to provide significant improvements in outcomes.

The outcomes for LAPC have remained poor and even with various schedules of radiotherapy, chemotherapy and even targeted agents, patients continue to have a grim outlook. Thus, LAPC with its fatal outcome is one of the ideal disease situations where HT in combination with CTRT needs to be evaluated in a randomized setting. Since most of the patients diagnosed with pancreatic cancer present as locally advanced disease (which are primarily inoperable), the proposed study is designed to explore the efficacy of local HT along with the conventional CTRT as a phase II randomized trial in LAPC.

An ongoing phase III trial, HEAT of adjuvant gemcitabine or gemcitabine with cisplatinum along with local HT has therefore been initiated as an adjuvant therapy in patients of resectable pancreatic cancers following a R0 or R1 resection [24]. To date, there has been no randomized study conducted or reported using HT in LAPC.

## Conclusion

The present phase II randomized study, HEATPAC with triple sensitization of concurrent HTCTRT along with pre-and post-interventional FOLFIRINOX could provide a potentially useful therapeutic option in LAPC. Based on the strong thermoradiobiological basis and its potentiation with concurrent CTRT, the outcomes are expected to be in favor with HT combined with CTRT. This could pave the way for a future phase III clinical trial in LAPC.

## Abbreviations

5-FU: 5-fluorouracil
CECT: Contrast enhanced computed tomography
CT: Chemotherapy
CTCAE: Common Terminology Criteria for Adverse Events
CTRT: Chemoradiotherapy
CTV: Clinical target volume
Dmax: Maximum dose
Dmin: Minimum dose
EGFR: Epidermal growth factor
ESHO: European Society of Hyperthermia Oncology
FOLFIRINOX: Folinic acid, Oxaliplatin, Irinotecan and 5-FU
GITSG: Gastrointestinal Tumor Study Group
GLOBOCAN: Global Cancer Incidence, Mortality and Prevalence
GTV: Gross target volume
HEAT: Hyperthermia European Adjuvant Trial
HT: Hyperthermia
HTCTRT: Thermochemoradiotherapy
JAMA: Journal of the American Medical Association
LAPC: Locally advanced pancreatic cancers
MRI: Magnetic resonance imaging
OS: Overall survival
PET: Positron emission tomography
PFS: Progression free survival
PTV: Planning target volume
RECIST: Response Evaluation Criteria in Solid Tumors
RT: Radiation
RTOG: Radiation Therapy Oncology Group
SIB-IMRT: Simultaneous-integrated boost intensity-modulated radiation therapy
WBC: White blood cell
